# Homogeneous Environmental Selection Structures the Bacterial Communities of Benthic Biofilms in Proglacial Floodplain Streams

**DOI:** 10.1128/aem.02010-22

**Published:** 2023-02-27

**Authors:** Jade Brandani, Hannes Peter, Stilianos Fodelianakis, Tyler J. Kohler, Massimo Bourquin, Grégoire Michoud, Susheel Bhanu Busi, Leila Ezzat, Stuart Lane, Tom J. Battin

**Affiliations:** a River Ecosystems Laboratory, Ecole Polytechnique Fédérale de Lausanne, Lausanne, Switzerland; b Department of Ecology, Faculty of Science, Charles University, Prague, Czechia; c Systems Ecology Group, Luxembourg Centre for Systems Biomedicine, University of Luxembourg, Esch-sur-Alzette, Luxembourg; d Group AlpWISE, Institute of Earth Surface Dynamics (IDYST), University of Lausanne, Lausanne, Switzerland; Norwegian University of Life Sciences

**Keywords:** proglacial floodplains, benthic biofilms, microbial diversity, 16S and 18S rRNA amplicons, climate change, assembly processes

## Abstract

In proglacial floodplains, glacier recession promotes biogeochemical and ecological gradients across relatively small spatial scales. The resulting environmental heterogeneity induces remarkable microbial biodiversity among proglacial stream biofilms. Yet the relative importance of environmental constraints in forming biofilm communities remains largely unknown. Extreme environmental conditions in proglacial streams may lead to the homogenizing selection of biofilm-forming microorganisms. However, environmental differences between proglacial streams may impose different selective forces, resulting in nested, spatially structured assembly processes. Here, we investigated bacterial community assembly processes by unraveling ecologically successful phylogenetic clades in two stream types (glacier-fed mainstems and non-glacier-fed tributaries) draining three proglacial floodplains in the Swiss Alps. Clades with low phylogenetic turnover rates were present in all stream types, including *Gammaproteobacteria* and *Alphaproteobacteria*, while the other clades were specific to one stream type. These clades constituted up to 34.8% and 31.1% of the community diversity and up to 61.3% and 50.9% of the relative abundances in mainstems and tributaries, respectively, highlighting their importance and success in these communities. Furthermore, the proportion of bacteria under homogeneous selection was inversely related to the abundance of photoautotrophs, and these clades may therefore decrease in abundance with the future “greening” of proglacial habitats. Finally, we found little effect of physical distance from the glacier on clades under selection in glacier-fed streams, probably due to the high hydrological connectivity of our study reaches. Overall, these findings shed new light on the mechanisms of microbial biofilm assembly in proglacial streams and help us to predict their future in a rapidly changing environment.

**IMPORTANCE** Streams draining proglacial floodplains harbor benthic biofilms comprised of diverse microbial communities. These high-mountain ecosystems are rapidly changing with climate warming, and it is therefore critical to better understand the mechanisms underlying the assembly of their microbial communities. We found that homogeneous selection dominates the structuring of bacterial communities in benthic biofilms in both glacier-fed mainstems and nonglacier tributary streams within three proglacial floodplains in the Swiss Alps. However, differences between glacier-fed and tributary ecosystems may impose differential selective forces. Here, we uncovered nested, spatially structured assembly processes for proglacial floodplain communities. Our analyses additionally provided insights into linkages between aquatic photoautotrophs and the bacterial taxa under homogeneous selection, potentially by providing a labile source of carbon in these otherwise carbon-deprived systems. In the future, we expect a shift in the bacterial communities under homogeneous selection in glacier-fed streams as primary production becomes more important and streams become “greener”.

## INTRODUCTION

Proglacial floodplains are dynamic landscapes that are substantially increasing in extent due to climate change (1). The transition from glacial to nonglacial cover has profound hydrological and biogeochemical impacts both locally on glacier margins and on downstream ecosystems (2, 3). However, proglacial floodplains continue to be under glacier influence (e.g., through the supply of meltwater, the melt-out of sediment-buried ice), and the recession of glacier ice leads to the establishment of biogeochemical and ecological gradients across small spatial scales. Driven by the time since ice melt, both lateral and longitudinal gradients may develop, with the latter often being expressed as chronosequences (4–6). Studying these environments in proglacial floodplains offers great opportunities to better understand the dynamics of microbial communities at the interplay between the biosphere, hydrosphere, and cryosphere.

Proglacial floodplains typically comprise a mainstem glacier-fed stream (GFS), often organized into a system of braided channels, and groundwater- or snowmelt-fed tributary streams (TRIBs) draining lateral hillslopes (7, 8). Compared to TRIBs, GFSs are characterized by a pronounced diel and seasonal variations in discharge, reduced channel stability, a permanently low streamwater temperature, and high turbidity (9–11). The high turbidity in GFSs results from the high loads of suspended mineral particles originating from the erosive activity of glaciers, affecting light availability and potentially also imposing abrasive forces on benthic communities (12). Despite these marked differences, both GFSs and TRIBs share environmental constraints, including low nutrient availability, low temperatures, and exposure to damaging UV radiation.

Over small spatial scales, streams within proglacial floodplains differ markedly in terms of water sources, hydrological regimes, and biogeochemical characteristics (13–15). This environmental heterogeneity results in a remarkable microbial diversity of biofilms coating benthic sediments (16, 17). While we are increasingly understanding the spatiotemporal patterns of microbial diversity in these streams, the relative importance of environmental constraints on these communities remains unknown. Previous work has highlighted compositional (18) and functional differences across GFSs and TRIBs; however, the processes that shape these communities remain unresolved.

The recent conceptual unification of community ecology (19) and methodological advances leveraging phylogenetic signatures and null model comparisons (20, 21) provided us with a framework to unravel such processes. In the context of proglacial floodplains, community assembly processes related to the introduction of novel taxa to local communities (i.e., dispersal in a metacommunity context [20]) may be particularly relevant regarding patterns of microbial biofilm development and the high interconnectivity of various habitats. High dispersal rates within and across proglacial habitats may be expected due to low sediment stability, fast flow, and high stream interconnectivity in braided parts of the floodplain. Stochastic processes are expected to dominate during the early phases of community establishment (22), mainly because of a lack of stabilizing interactions among community members. On the other hand, previously reported differences in the diversity and composition of TRIB and GFS biofilm communities (18) point toward the role of selection as an important process. Selection can be the consequence of the abiotic environment and interactions among community members. Across the various habitats of proglacial floodplains, environmental constraints related to nutrient scarcity and low temperatures may lead to homogenizing selection (23, 24). However, important differences between GFS and TRIB ecosystems may impose differential selective forces.

In this work, we first identify processes governing the assembly of benthic biofilm communities in GFSs (*n* = 128 sites) and TRIBs (*n* = 125 sites) of three proglacial floodplains in the Swiss Alps. Given the multiplicity of environmental constraints in these ecosystems and based on previous work on GFSs in New Zealand (24), we expect that homogeneous selection (HoS) dominates community assembly in proglacial streams. We then used a recently developed analytical tool, phyloscore (24), to explore which bacterial clades contribute to the community-wide assembly processes in GFSs and TRIBs. We expect that clades under selection in both TRIBs and GFSs reflect the overarching environmental constraints of proglacial environments, whereas clades with signatures of selection in only one of the stream types reflect nested, specific constraints. Indeed, consistent environmental differences between GFSs and TRIBs impose different selective forces, and hence, we expect to observe nested and spatially structured assembly processes for proglacial floodplain communities. Finally, leveraging the chronosequences of proglacial floodplains, we hypothesize that stochastic processes dominate toward more recently deglaciated areas (24). Understanding the nature of these constraints and their impacts on microbial communities in these ecosystems is particularly relevant in light of climate change and the expected predominance of TRIBs in the future. In line with this, our previous work indicated an important role of eukaryotic phototrophs in explaining the differences between GFS and TRIB communities (18, 25, 26). We expect that organic carbon provided by benthic primary producers can alleviate (to some extent) resource limitation in TRIBs and, thus, reduce the importance of selection therein. Finally, by studying proglacial floodplain chronosequences, we assess whether similar assembly processes extend across proglacial floodplains.

## RESULTS AND DISCUSSION

### Homogeneous selection dominates community assembly.

We sampled benthic sediments in GFSs and TRIBs from the Otemma Glacier (OTE), Val Roseg Glacier (VAR), and Valsorey Glacier (SOY) floodplains and explored bacterial communities. We applied a community-level framework (20, 21) to analyze processes governing community assembly within each stream and floodplain. Briefly, abundance-weighted phylogenetic clustering (i.e., lower across-sample phylogenetic distance than expected by null simulations [β-nearest-taxon index {βNTI}]) is interpreted as signatures of similar environmental conditions selecting for phylogenetically similar taxa across the sample pair (i.e., homogeneous environmental selection). In contrast, no deviation from the null expectation or phylogenetic overdispersion would indicate the importance of stochastic processes or heterogeneous environmental selection, respectively.

**FIG 1:**
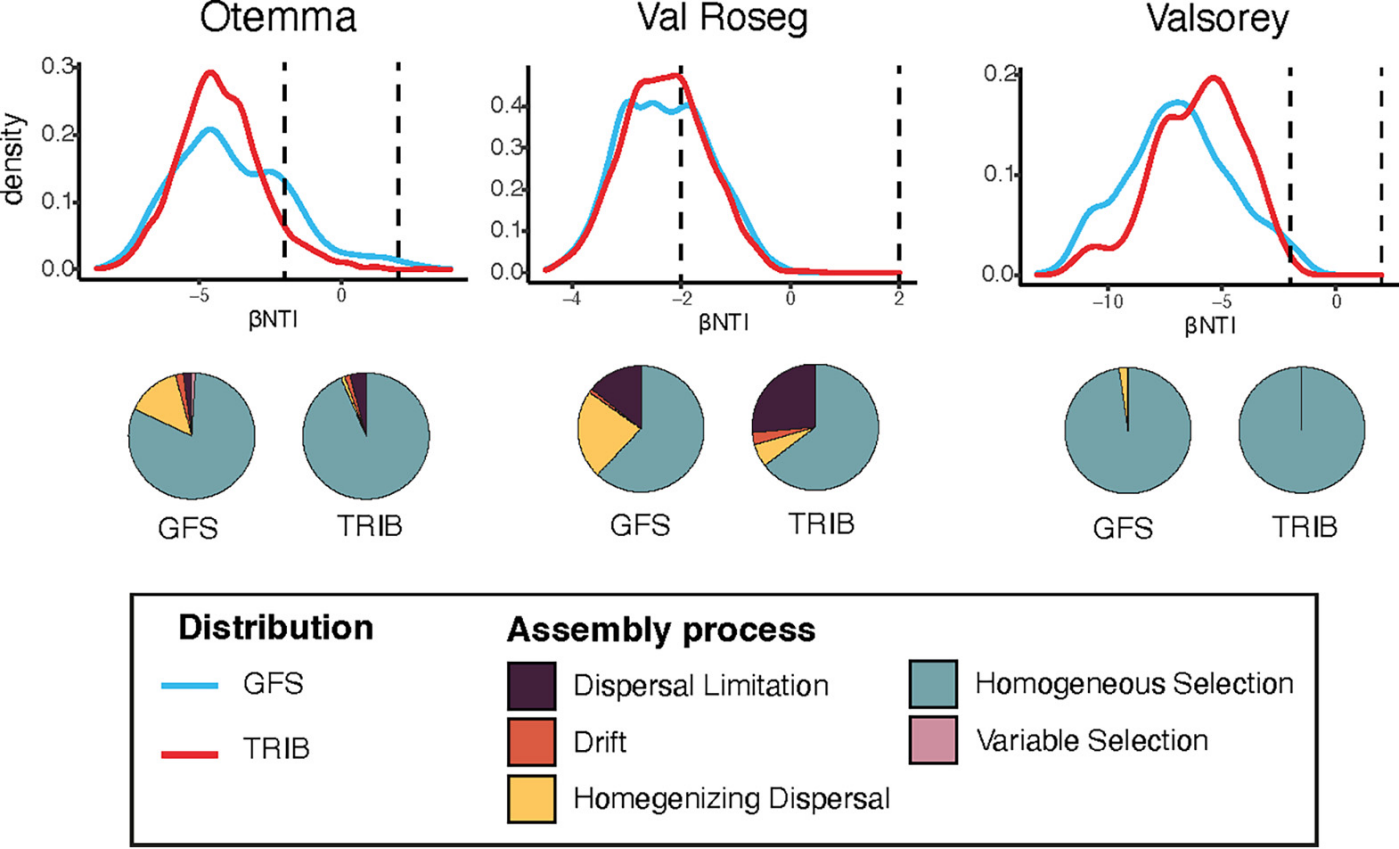
Homogeneous selection is the dominant assembly process in both streams and all three proglacial floodplains. The density plots show the probability density curves for β-nearest-taxon index (βNTI) values based on 16S rRNA for sample comparisons within glacier-fed streams (GFS) and within tributary streams (TRIB) sampled for the three glacier floodplains. The *y* axis represents the probability of the βNTI values, and the area under each curve is equal to 1. Vertical dashed lines represent the −2 and 2 βNTI thresholds, which are the cutoff values for lower-than-expected (homogeneous selection) and higher-than-expected (variable selection) phylogenetic community turnover rates. The assembly processes governing the sample pairs in between are estimated from compositional turnover patterns based on RC_Bray_. Pie charts display the respective proportions of assembly processes.

We found homogeneous selection (HoS) to be the dominant assembly process within GFSs and TRIBs for all three proglacial floodplains (81.1% of community pairs in GFSs from OTE [GFS_OTE_], 93.4% in TRIB_OTE_, 62.0% in GFS_VAR_, 64.7% in TRIB_VAR_, 97.7% in GFS_SOY_, and 100% in TRIB_SOY_) (Fig. 1). HoS was recently reported to be the dominant assembly process in GFS microbial biofilms (23, 24) and other energy-limited ecosystems (27–29). The extreme conditions in GFSs (i.e., the permanently low temperature, the ultraoligotrophic environment, abrasion by suspended particles, and sediment instability) may jointly contribute to the selection forces leading GFS microbial communities to assemble deterministically. In contrast, we predicted that increased habitat stability and enhanced carbon and nutrient availability mediated by the presence of primary producers would weaken the selective force in TRIBs. Together with additional niches, putatively linked to phototrophic-heterotrophic interactions (26), we expected an increased importance of heterogeneous assembly across TRIBs. However, this was not the case. Instead, similarly to those of GFSs, TRIB bacterial communities were governed primarily by HoS, suggesting that similar environmental constraints (within TRIBs) shape these communities. This is in contrast to the notion of subtle environmental gradients along lateral dimensions of proglacial floodplains and rather suggests that as glaciers retreat, stream communities shift from one deterministic domain (i.e., GFSs) to another deterministic domain (i.e., TRIBs).

While HoS was the dominant process across all floodplains and both stream types, the relative contribution of HoS in VAR was lower than those in OTE and SOY streams. Indeed, βNTI distributions for VAR (Fig. 1) indicated that a substantial fraction of the sample pairs showed signs of stochastic community assembly.

### Nested homogeneous selection.

While our initial community-level analysis revealed that HoS is the dominant force shaping microbial communities in GFSs and TRIBs, we expected different bacterial clades to contribute to these community-wide signatures. To address this, we used phyloscore analysis to identify mono- or paraphyletic clades with significant signs of phylogenetic clustering. Such clades under homogeneous environmental selection were previously identified to dominate GFS communities and disproportionately contribute to local diversity through microdiversification (24). Here, we identified 18 HoS clades (i.e., clades with significantly lower phyloscores than those of the outgroups [6.9e−242 < *P* < 1.37e−19 by contrast tests]) across all proglacial floodplains and streams. These clades contained between 22 and 2,280 amplicon sequence variants (ASVs) (Table 1). Out of all identified clades, the consensus taxonomy for 12 of them was associated with the *Proteobacteria* phylum at various taxonomy levels such as *Alphaproteobacteria* (clades 5, 7, and 11), *Burkholderiales* (clades 1, 4, 10, and 17), *Comamonadaceae* (clades 9 and 16), and *Rhodoferax* (clade 3) (Table 1). Other identified clades were affiliated with *Vicinamibacteraceae* (clades 2, 8, 13, and 18), and one clade comprising 659 ASVs in TRIB_VAR_ was affiliated with *Chitinophagaceae*. Similar to a previous study by Fodelianakis et al. (24), we did not observe phylogenetic clades with significantly higher total phyloscores, which further emphasizes the low contribution of heterogeneous selection to governing community assembly (Fig. 1).

**TABLE 1:** Phylogenetic clades under homogeneous selection for each proglacial floodplain (i.e., OTE, VAR, and SOY) and each stream (i.e., GFSs and TRIBs)^*a*^

Glacier	Stream type	Clade	No. of ASVs	Consensus taxonomy	Associated phylum
OTE	GFS	1	1,639	*Burkholderiales*	*Proteobacteria*
		2	64	*Vicinamibacteraceae*	*Acidobacteriota*
		3	28	*Rhodoferax*	*Proteobacteria*
	TRIB	4	1,860	*Burkholderiales*	*Proteobacteria*
		5	1,064	*Alphaproteobacteria*	*Proteobacteria*
VAR	GFS	6	2,372	*Proteobacteria*	*Proteobacteria*
		7	1,123	*Alphaproteobacteria*	*Proteobacteria*
		8	73	*Vicinamibacteraceae*	*Acidobacteriota*
		9	22	*Comamonadaceae*	*Proteobacteria*
	TRIB	10	2,280	*Burkholderiales*	*Proteobacteria*
		11	1,373	*Alphaproteobacteria*	*Proteobacteria*
		12	659	*Chitinophagaceae*	*Bacteroidota*
		13	82	*Vicinamibacteraceae*	*Acidobacteriota*
SOY	GFS	14	2,172	*Proteobacteria*	*Proteobacteria*
		15	264	*Vicinamibacteraceae*	*Acidobacteriota*
		16	26	*Comamonadaceae*	*Proteobacteria*
	TRIB	17	1,151	*Burkholderiales*	*Proteobacteria*
		18	220	*Vicinamibacteraceae*	*Acidobacteriota*

a The numbers of ASVs within each clade are reported. All *P* values by contrast tests were <0.001.

**FIG 2:**
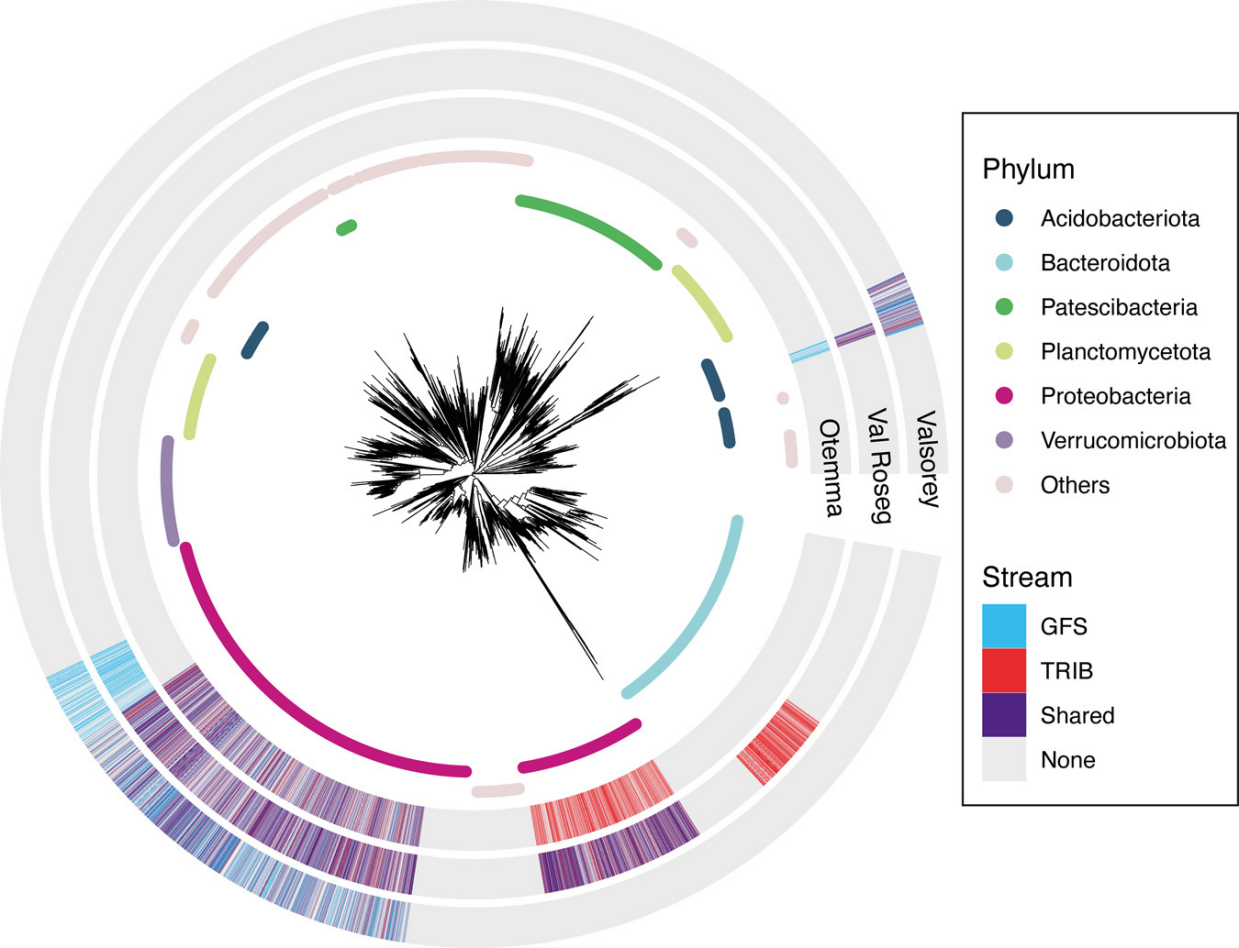
HoS ASVs are diverse, and differences emerge between streams and across the three proglacial floodplains. The identified 16S rRNA ASVs are part of phylogenetic clades under homogeneous ecological selection (HoS clades) with >20 ASVs. The respective consensus taxonomies are color-coded on the inner ring at the phylum level. ASV presence is compared between GFS and TRIB sites and is color-coded on the outer rings for each of the three glacial floodplains.

After identifying HoS clades for the different categories (GFSs and TRIBs for each proglacial floodplain), we compared the presence/absence of ASVs within HoS clades within each proglacial floodplain and between stream types. We found 3,240, 4,928, and 2,594 unique ASVs under HoS for OTE, VAR, and SOY, respectively, but observed different patterns regarding the exclusive or shared presence between stream types. For OTE, 9.7% and 47.4% of ASVs under HoS were found exclusively in GFSs and TRIBs, respectively. The remaining 42.8% of ASVs in HoS clades were present in both stream types in OTE. These shared ASVs were classified as *Gammaproteobacteria* (e.g., *Burkholderiales*), while *Alphaproteobacteria* encompassed clades present exclusively in TRIBs (Fig. 2 and Table 1). For VAR, we observed an even higher percentage of shared ASVs (i.e., 61.6%) affiliated with *Acidobacteriota*, *Gammaproteobacteria*, and *Alphaproteobacteria*. We observed a subclade present exclusively in GFS_VAR_ (10.8%) associated with *Comamonadaceae* and a *Chitinophagaceae* clade present exclusively TRIB_VAR_ (27.6%) (Fig. 2). Finally, for SOY, we found that 48% of HoS ASVs were present exclusively in GFSs, while 8.1% were exclusive to TRIBs, and 43.9% were shared.

Taken together, the substantial overlap of clades under selection suggests a common selective force on GFSs and TRIBs across all floodplains. At the same time, distinct (also taxonomically) HoS clades were found in both stream types across the three floodplains, supporting the notion of nested selective forces within these floodplains. While several assembly processes act in concert on any microbial community, we suspect that such nested and clade-specific assemblies may be common among spatially close but environmentally different communities, such as, for instance, rock pools (30). This finding is also important when considering the impacts that climate change will have on proglacial streams, where the reduction of glacial meltwater inputs will render GFSs more similar to TRIBs (15, 17, 31). We suggest that clades under homogeneous selection will play a key role in the reassembly of proglacial communities in the future. We expect that overarching selective forces, such as low water temperatures, a scarcity of organic and inorganic nutrients, a short ice-free season, and high levels of UV radiation, will change much less than stream-specific environmental forces, which are tightly related to the presence of the glacier (e.g., high turbidity, pronounced diurnal variation in flow, and unstable streambed habitats). Based on this, we would predict a shift toward increasing prevalences of *Burkholderiales*, *Alphaproteobacteria*, and *Vicinamibacteraceae* in future GFSs. Moreover, HoS clades, per definition, are characterized by high phylogenetic clustering, implying that a large number of phylogenetically closely related taxa are already present in different proglacial habitats. Such regional microdiversity may allow these clades to more readily occupy new niches when the selective forces of GFSs approximate those within TRIBs. This notion is supported by the substantial contributions of HoS clades to alpha diversity. Specifically, HoS clades accounted for 32.9% of ASV richness in each sample on average, with a slightly higher relative contribution in GFSs (average, 34.8% ± 7.5%) than in TRIBs (average, 31.1% ± 9.2%) (*P* < 0.001 by a *t* test). Similarly, HoS clades also dominated proglacial stream abundances, accounting for 56% ± 13.9% of the relative abundance on average. The relative abundance of HoS clades was higher in GFSs (61.3%) than in TRIBs (50.1%) (*P* < 0.001 by a *t* test). We thus predict that the shift from GFSs toward TRIBs induced by climate change will lead to an increase in local biodiversity through an expansion of HoS clades and a reduction of dominance (i.e., an increase in community evenness).

### Role of photoautotrophic biomass in community assembly.

**FIG 3:**
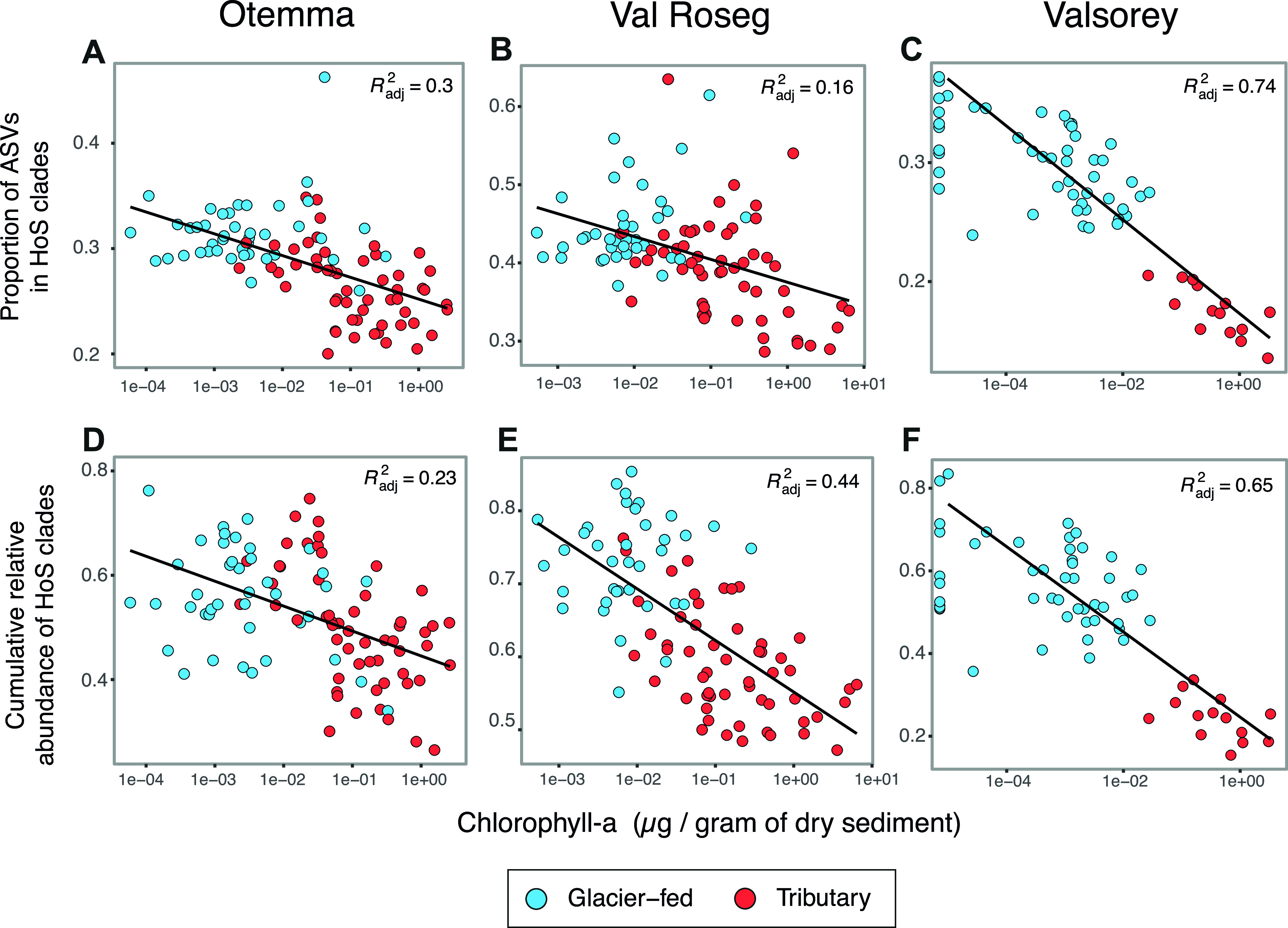
HoS clades thrive in sediments with low chlorophyll *a* concentrations, in both abundance and alpha diversity. (A to C) Proportion of ASVs in HoS clades as a function of the sediment chlorophyll *a* concentration for Otemma (A), Val Roseg (B), and Valsorey (C). (D to F) Cumulative relative abundance of HoS clades as a function of the sediment chlorophyll *a* concentration for Otemma (A), Val Roseg (B), and Valsorey (C).

**FIG 4:**
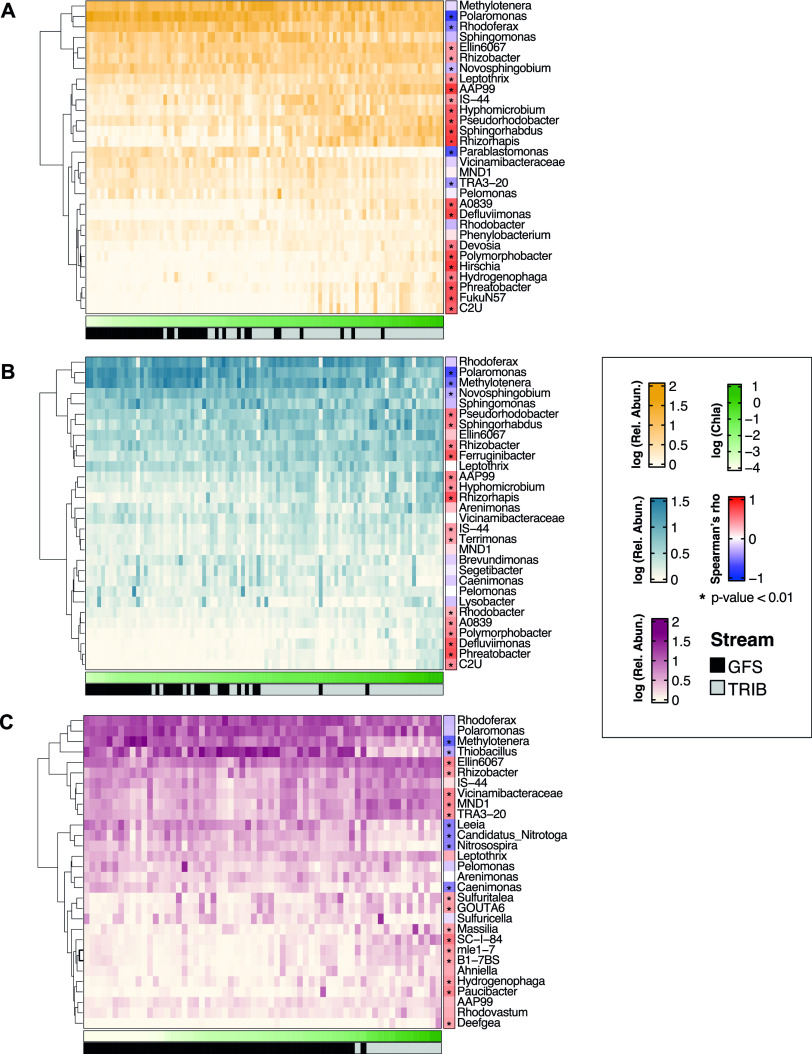
Photoautotrophic biomass structure taxa under homogeneous selection. Heat maps for Otemma (A), Val Roseg (B), and Valsorey (C) display the relative abundances of ASVs under homogeneous selection at the genus level. Sites are ordered by chlorophyll *a* (Chla) concentrations, and the respective streams are displayed in the horizontal bars. On the right, the Spearman correlation of genera and chlorophyll *a* is displayed, and asterisks indicate a significant correlation.

In proglacial streams, benthic primary production is strongly affected by streamwater turbidity (18, 32–34), with important cascading effects on microbial heterotrophic production in these otherwise organic-carbon-deprived ecosystems (16, 35). Therefore, we explored the role of photoautotrophic biomass in the structuring of bacterial taxa under HoS. To achieve this, we tested for relationships of both alpha diversity and the abundance of HoS clades with chlorophyll *a* concentrations. We found that both HoS clade abundance and diversity decreased disproportionately compared to the rest of the microbiome as sediment chlorophyll *a* increased (linear models, *P* < 0.001 for all models; adjusted *R*^2^, 0.16 to 0.74) (Fig. 3). We next explored the relationships between specific HoS clades (relative abundance at the genus level) and chlorophyll *a* concentrations and found significant negative correlations for *Methylotenera*, *Polaromonas*, *Rhodoferax*, and *Thiobacillus* in all floodplains (Fig. 4). These genera have been repeatedly found in GFSs (23, 24, 36) and other cryospheric ecosystems (37, 38) and are known to engage in methylotrophic, mixotrophic, and chemolithoautotrophic metabolism. In contrast, the relative abundances of *Rhizobacter*, Ellin6067, and *Sphingomonas*, besides several other members of HoS clades, increased significantly with increasing chlorophyll *a* concentrations (Fig. 4). Therefore, we suggest that, among other selective environmental conditions, organic carbon availability linked to the presence of phototrophs exerts selective forces on proglacial streams. This is particularly relevant considering the successional dynamics of proglacial floodplains. While key environmental constraints, such as temperature, may respond relatively directly to reduced glacial influence (13), the establishment of algal communities likely follows nonlinear successional trajectories, putatively characterized by a transition from low-diversity communities dominated by pioneer algal species to more diverse algal communities (34). Superimposed on this spatial trend will be progressive reductions in glacial erosion as glaciers thin and retreat and, hence, suspended sediment supplies and turbidity, and while the mechanism of selection (e.g., resource availability) may exert deterministic selective forces, we suspect that stochasticity associated with photoautotrophic community succession will imprint future microbial community assembly in proglacial floodplains.

### Chronosequence succession effect on taxa under homogeneous selection.

**FIG 5:**
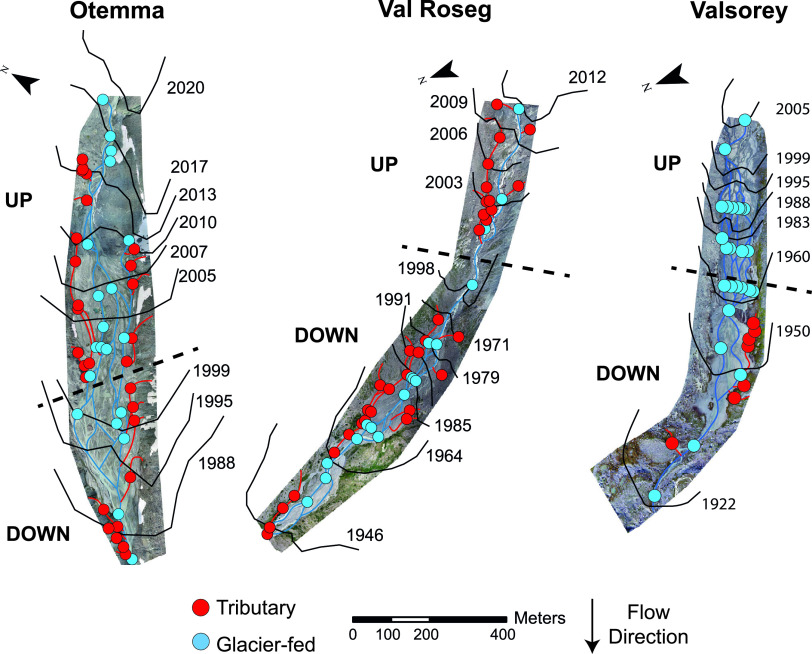
Maps of the proglacial floodplains displaying sampling sites and glacier extents from 1922 to 2020. Sites are colored according to their stream type. The black solid lines represent the maximum glacial extent in the given year and were collected from historic maps and orthophotos (see Materials and Methods for additional details). The black dashed lines display the split between the upper region (UP) (i.e., close to the glacier’s snout) and the downstream regions (DOWN).

To substantiate the notion of successional imprints on microbial assembly, we opted for a space-for-time substitution approach (e.g., see reference 39), where we compared assembly processes of older (i.e., longer time since glacier melt) and younger (shorter time since glacier melt) regions in the floodplains. We arbitrarily split the floodplains into downstream (DN) and upstream (UP) regions (Fig. 5) and identified assembly processes within each chronosequence region and floodplain as described above. Strikingly (and contrary to our expectations), we did not find differences in the processes governing assembly at the community level between UP and DN sites. In both regions and all floodplains, HoS was the overall dominating process (ranging from 58.9% to 100% of sample pairs) (see Fig. S1 in the supplemental material). Resolving clades that contribute to signatures of HoS in these ecosystems, we found that several HoS clades were present in both UP and DN regions, whereas few clades were identified exclusively in one region or the other. For instance, in OTE, *Alphaproteobacteria* exhibited signatures of homogeneous selection in DN regions in GFSs, whereas *Bacteroidota* were exclusively under HoS in UP regions in TRIBs (Fig. S2). However, these patterns were not consistent across the different floodplains. We argue that despite the several decades since glacier retreat, key environmental constraints remain relatively unchanged across the floodplains, particularly for GFSs. In line with this, we could not detect statistically significant differences in measured environmental parameters (e.g., temperature, pH, nutrients, and conductivity) between UP and DN regions (Table S1). While the dominant environmental constraints in GFSs may extend far downstream, we suggest that environmental conditions in TRIBs and, hence, the dominant selective forces acting on microbial communities therein are already in place in the uppermost, groundwater-fed tributary streams.

### Conclusion.

Our results show that across three proglacial floodplains, benthic biofilm community assemblies within GFSs and TRIBs are both largely dominated by homogeneous selection. While this confirms our initial hypotheses, we expected to see stronger differences in the relative contributions of stochastic processes between GFSs and TRIBs. We identified similar ecologically successful phylogenetic clades across each stream, suggesting a common driver of GFS and TRIB microbiome assembly across all floodplains. At the same time, distinct HoS clades were found in both types of streams across the three floodplains, supporting the notion of nested selective forces within these floodplains. Additionally, our analysis provides first insights into the correlation between aquatic photoautotrophic biomass and bacteria under homogeneous selection, by potentially providing a labile source of carbon in these otherwise carbon-deprived systems. As glaciers shrink, proglacial environments will shift from glacier melt-dominated to groundwater-dominated environments. Hence, this study provides a more complete understanding of the identity of phylogenetic clades likely to be lost under a changing climate. Further large-scale studies (>1 km) are required to better capture the temporal ontogeny of these environments and assess the impact of time since deglaciation on the success of microbial communities.

## MATERIALS AND METHODS

### Study sites and sample collection.

We collected 253 sediment samples from streams draining three proglacial floodplains in the Swiss Alps, Otemma (OTE) (45°56′08.4″N, 7°24′55.1″E), Val Roseg (VAR) (46°24′21.1″N, 9°51″55.1″E), and Valsorey (SOY) (45°55′09.4″N, 7°15′34.2″E), over the summer of 2019. Sampling occurred twice, early (i.e., late June/early July) and later (late August/early September) in the glacier melt season. Samples from both seasons were included, as previously reported analyses have shown no difference in bacterial communities or the biomasses between early and late seasons (18). Study sites included both glacier-fed streams (GFSs) and nonglacial lateral tributary streams (TRIBs) located near the glacier snout and continuing to the proglacial floodplain’s outlet, with a maximum distance ranging from 1,000 to 1,300 m. We collected totals of 42, 38, and 48 GFS samples and totals of 55, 56, and 14 TRIB samples for OTE, VAR, and SOY, respectively (Table 2). Within each sampling site, we collected from the benthic zone (~5 cm in the streambed) sandy sediment (0.250 to 3.15 mm) using flame-sterilized sieves and spatulas. Samples (~10 g) for the analysis of chlorophyll *a* and DNA extraction were transferred to sterile cryovials and flash-frozen on dry ice immediately after collection. Additional details on sampling and the glacier floodplains’ physicochemical characteristics were previously reported (18).

**TABLE 2:** Floodplain characteristics and sampling design^*a*^

Parameter	Value for site		
	Otemma	Val Roseg	Valsorey
Altitude (m above sea level) (range)	2,400–2,600	2,090–2,340	2,400–2,500
Floodplain length (m)	1,300	1,100	1,000
Avg floodplain slope (%)	15.3	22.7	10
Floodplain width (m) (range)	50–160	50–250	30–90
Early sampling date (days and mo)	8–10 July	2–3 July	24–26 June
Late sampling date (days and mo)	21–22 August	10–11 September	17–18 September
Total no. of GFS samples	42	38	48
GFS UP	29	6	26
GFS DN	13	32	22
Total no. of TRIB samples	55	56	14
TRIB UP	36	22	0
TRIB DN	19	34	14

Total no. of samples	97	94	62

a Data from reference 18.

We also collected streamwater samples from a subset of the GFS and TRIB study reaches due to logistical constraints, as reported previously by Brandani et al. (18). Briefly, streamwater turbidity was monitored using PME Cyclops-7 loggers (every minute) and averaged over the sampling duration. We measured streamwater conductivity using a WTW-IDS probe (TetraCon 925) and pH, temperature, and dissolved oxygen using a WTW multiparameter portable meter (MultiLine Multi 3630 IDS). Streamwater samples for the determination of dissolved organic carbon (DOC) were filtered (precombusted GF/F filters; Whatman, UK) and stored in acid-rinsed and precombusted glass vials at 4°C. Samples for the analysis of major ions were filtered (0.22-μm Sterivex filters [Millipore] with a Durapore polyvinylidene difluoride [PVDF] membrane) and stored at 4°C. Finally, aliquot samples for the measurement of inorganic nutrients were collected in acid-washed 30-mL Nalgene high-density polyethylene (HDPE) bottles, flash-frozen on dry ice, and stored at −20°C.

### Glacial retreat and proglacial floodplain development.

Proglacial floodplains were formed with glacial retreat over time, and the maximum extent of the glacier’s ice for a given year was recorded regularly. We collected former glacial extents for each floodplain to assess the time since deglaciation for each study site. To achieve this, we first gathered former glacier extents from historic maps and orthophotos using the SWISSIMAGE journey through time online tool (40) and the GLIMS glacier inventory (41). In addition, these extents were carefully compared with glacier monitoring in switzerland (GLAMOS) (42) frontal variation measurements to verify glacial readvance. The fine-scale sampling effort on the three proglacial floodplains allowed the use of a time-for-space substitution to address the question of proglacial floodplain temporal ontogeny by comparing the upper region of the floodplain (UP) (i.e., close to the glacier’s snout) to the downstream region (DN). The rationale behind this is that the floodplain’s DN region has been deglaciated for a longer period than the UP region and may serve as an example of a more stable region where biofilm communities have grown for a longer time. Specifically, for the study sites, the latest glacial extents for the DN region ranged from 1988 to 2000, 1946 to 2000, and 1922 to 1960 for OTE, VAR, and SOY, respectively. For the UP region, the latest glacial extents ranged from 2001 to 2019, 2001 to 2012, and 1961 to 2005 for OTE, VAR, and SOY, respectively (Fig. 5).

### Streamwater solute analyses.

Inorganic nutrients (ammonium, nitrite, nitrate, and soluble reactive phosphorus) were determined using a LaChat QuikChem 8500 flow injection analyzer. The concentration of DOC was measured using a Sievers M5310c total organic carbon (TOC) analyzer (GE Analytical Instruments) (accuracy, ±2%; precision, <1%; detection limit, 22 μg C/L).

### Bacterial abundance.

Bacterial abundance (cells per gram of dry sediment) was estimated using flow cytometry (24, 32) according to methods described previously by Brandani et al. (18). Detachment of cells from sediments was achieved by mild shaking (standard analog shaker; VWR) (15 min at speed 5.5) and sonication (Sonifier 450; Branson) (1 min, 60% duty cycle, and output 5) in 10 mL of a paraformaldehyde-glutaraldehyde solution supplemented with sodium pyrophosphate (final concentration of 0.025 mM). The supernatant was diluted and stained with SYBR green (1× final concentration) (incubation for 15 min at 37°C) before analysis on a flow cytometer (NovoCyte; Acea Biosciences) equipped with a 488-nm laser. We analyzed three stained technical replicates and one unstained replicate for each sample, and the coefficient of variation among technical replicates averaged 4.93% ± 3.84%.

### Chlorophyll *a*.

The sediment chlorophyll *a* concentration was measured according to a modified ethanol (EtOH) extraction protocol described previously (32). Briefly, 5 mL of 90% EtOH was mixed with ~2 g of wet sediment, extracted in a hot water bath (78°C for 10 min), and incubated in the dark (4°C) for 24 h. Following a vortex-and-centrifugation step, the supernatant was then read on a plate reader at 436/680 nm (excitation/emission wavelength). Using spinach chlorophyll *a* as a standard, chlorophyll *a* concentrations were quantified and are reported as micrograms of chlorophyll *a* per gram (dry mass [DM]) of sediment.

### DNA extraction, metabarcoding library preparation, and sequencing.

A modified phenol-chloroform method (43) was used to extract DNA, and all DNA samples were diluted to a final concentration of ≤2 to 3 ng/μL. Prokaryotic metabarcoding libraries targeting the V3-V4 hypervariable region of the 16S rRNA gene with the 341F/785R primer pair were prepared according to the manufacturer’s guidelines. Amplifications were verified on a 1.5% agarose gel, and a second PCR was performed to add dual indices to the purified amplicon PCR products. This allowed the multiplexing of samples on a single sequencing lane of the MiSeq platform (Illumina) after quantification and normalization. Samples were sequenced using a 300-base paired-end protocol at the Lausanne Genomic Technologies Facility (LGTF) (Switzerland).

### Downstream sequence analyses.

We used a combination of Trimmomatic v.0.36 (44) for quality filtering of sequencing reads and QIIME2 v.2020.8 (45). We further used DADA2 (46) with default parameters to remove primers and noise as well as to join reads into exact amplicon sequence variants (ASVs). We used the SILVA v.138.1 database (47) for taxonomic classification. A total of 253 amplicon sequence libraries were produced, and paired-end sequencing generated a total of 32,186,859 reads (18). Nonbacterial ASVs, including those affiliated with archaea, chloroplasts, and mitochondria, were discarded from the 16S rRNA amplicon data set. Singletons and ASVs observed at fewer than two sites from the same proglacial floodplain were also discarded. For each sample, we calculated the relative abundance of ASVs and multiplied this value by the bacterial abundance to obtain the ASV absolute abundance.

### Quantification of the dominant assembly processes at the community level.

The processes underlying community assembly include selection (either homogeneous or variable), dispersal (homogenizing or limiting), and drift. To quantify the relative contributions of community assembly processes within these two metacommunities (GFSs and TRIBs) for each proglacial floodplain, rates of phylogenetic and compositional turnover at the community level were quantified using the framework developed previously by Stegen et al. (20, 21). First, investigating the community-wise phylogenetic turnover between any two communities allows the assessment of the influence of selection. The β-mean-nearest-taxon distance (βMTD) is computed as the abundance-weighted mean of the nearest-taxon distances for taxa present in only one of the two compared communities by using the comdistnt function of the picante package (48) in R (setting abundance.weighted = TRUE). The β-nearest-taxon index (βNTI) refers to the z-score of the observed βMTD from a null distribution built for the same community pairs by shuffling phylogenetic tips, making the abundances and presence/absence of ASVs random in the compared communities but keeping the distribution of the phylogenetic distances.

βNTI scores lower than −2 indicate lower phylogenetic turnover rates than expected by chance and that homogeneous selection between the compared communities leads to higher-than-expected phylogenetic similarity (20, 21). Similarly, βNTI scores higher than +2 revealed the dominance of variable selection. However, community pairs with absolute βNTI (|βNTI|) values lower than +2 were further analyzed in terms of compositional turnover using Raup-Crick distances based on Bray-Curtis similarity (RC_Bray_). The rationale is that passive dispersal via abiotic factors (in this example, streamflow) should be blind to the taxon’s phylogeny and result in lower or higher compositional turnover rates (if dispersal is homogenizing or limiting, respectively). An RC_Bray_ value lower than −0.95 indicates a lower compositional turnover rate than the null expectation and is attributed to homogenizing dispersal. Likewise, an RC_Bray_ value higher than +0.95 is indicative of dispersal limitation (20, 21).

### Identification of phylogenetic clades and ASVs under homogeneous selection.

After inferring community-wide assembly patterns, we used a framework developed previously by Fodelianakis et al. (24) to identify clades under homogeneous selection (HoS clades). HoS clades are defined as groups with distinctly low clade-wise phylogenetic turnover rates, thus containing ASVs with phylogenetically closer relatives across communities than expected by chance. A z-score quantifies the difference of the nearest phylogenetic distance to the null distribution (phyloscore) for a given pair of communities and a given ASV. We further compute each ASV’s total phyloscore as the sum of its phyloscores across all community pairs. We use phylofactorization (49, 50) to identify clades of ASVs with a significantly different phyloscore than those of the outgroups and then extract the consensus taxonomic classification. This phyloscore framework identifies phylogenetic clades with lower phyloscores than those of the outgroups, often exposing high niche occupancy and homogeneous selection pressures. Additional methodology and algorithm details have been reported previously (24). With HoS clades identified for a given community (GFS or TRIB), the list of ASVs present in each HoS clade (HoS ASV) is compared to the list of the other communities within a given floodplain (i.e., Otemma GFS versus Otemma TRIB). HoS ASVs are then further categorized depending on whether their presence was found exclusively in GFSs or TRIBs or whether the same ASV was found to be under homogeneous selection in both communities and are referred to as “shared” (Fig. 2). ASVs identified as “none” were found in the sequencing data but were not identified in any phylogenetic clades under homogeneous selection.

Abundance patterns of HoS ASVs were further investigated with heat maps using the ComplexHeatmap R package (51). To observe the effect of photoautotrophic biomass on HoS ASVs, samples were reordered based on their chlorophyll *a* concentrations. ASVs were combined at the genus level using the tax_gloom function from the phyloseq package (52). For each floodplain, the 30 genera with the highest relative abundances in HoS clades are displayed. All statistical analyses were performed in R 4.0.3 (53).

We also used a space-for-time substitution to explore proglacial floodplain temporal ontogeny and compared the upper half of the floodplain (UP) to the lower half (DN). Indeed, the floodplain’s DN region has been deglaciated for a longer time than the UP region. Similarly, we depicted community assembly processes and identified HoS ASVs between UP and DN regions of proglacial floodplains within each stream (i.e., GFS and TRIB) and for individual floodplains.

### Data availability.

Sequencing data have been uploaded to the National Center for Biotechnology Information database under BioProject accession number PRJNA808857. Metadata and raw sequences used for the statistical analyses in this study have been deposited in the Zenodo repository under accession number 6424496 (https://doi.org/10.5281/zenodo.6424496).
