# Supplementary material for: Homogeneous Environmental Selection Structures the Bacterial Communities of Benthic Biofilms in Proglacial Floodplain Streams
Source: Appl Environ Microbiol. 2023 Feb 27;89(3):e02010-22. doi: 10.1128/aem.02010-22 (PMC10053691; doi:10.1128/aem.02010-22)
Supplement: Supplemental file 1 — Supplemental material. Download aem.02010-22-s0001.pdf, PDF file, 9.2 MB [file aem.02010-22-s0001.pdf]

Supplementary Material

1. Supplementary Figures

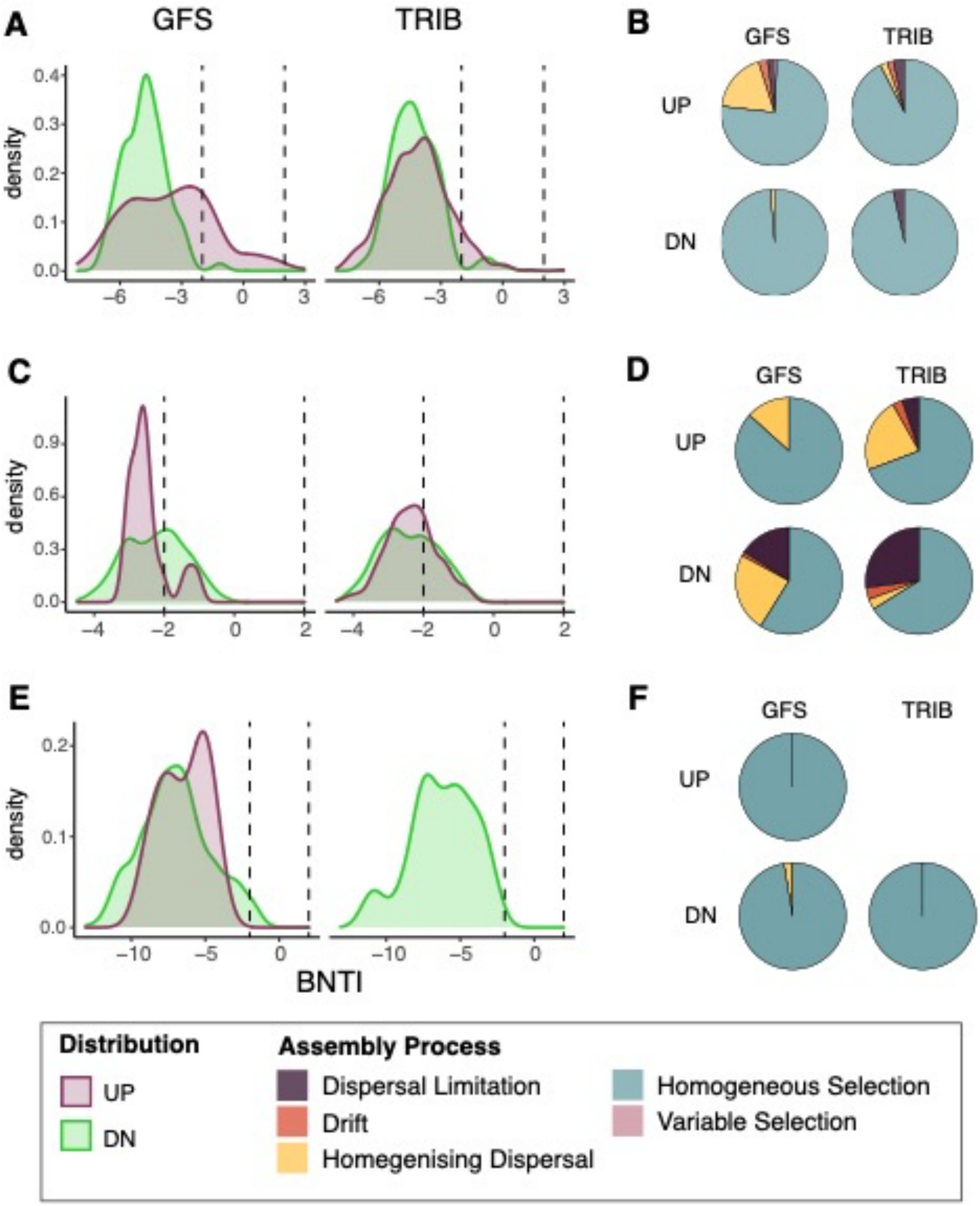

Figure S1: Homogeneous selection is the dominant assembly process in both UP and DN region for both stream types and all three proglacial floodplains. β-Nearest taxon Index (βNTI) distribution based on 16S rRNA for glacier-fed (GFS) and tributary (TRIB) streams

sampled for the three glacier floodplains (A: Otemma; C: Val Roseg; E: Valsorey). The dashed lines represent the -2 and 2 threshold depicting homogeneous selection and variable selection, respectively. Pie charts display the respective proportion of assembly processes for each floodplain (B: Otemma; D: Val Roseg; F: Valsorey).

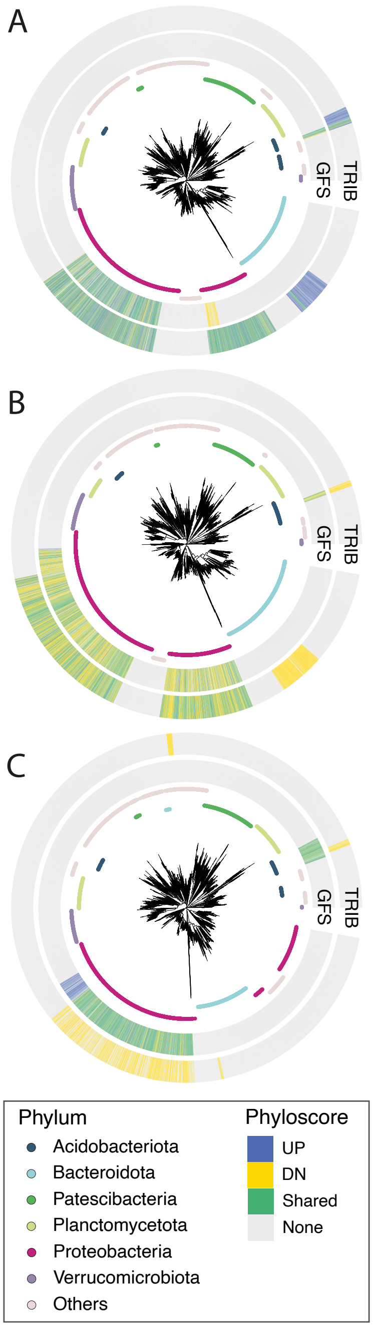

Figure S2: HoS ASVs are diverse, and differences emerge between stream type and regions within proglacial floodplains (A: Otemma, B: Val Roseg, C: Valsorey). Identified 16S rRNA ASVs, part of phylogenetic clades under homogeneous ecological selection (HoS clades) with > 20 ASVs. The respective consensus taxonomy is color-coded on the inner ring

at the phylum level. ASVs' presence/absence are compared between UP and DN sites for each stream type and proglacial floodplain and color-coded on the outer rings for each of the three glacial floodplains.

2. Supplementary Tables

Table S1. Streamwater physiochemical indicators for tributary and glacier-fed streams for downstream (DOWN) and upper region (UP) (mean ± standard deviation). A Welch Two sample t-test was used to test the significance between DOWN and UP region. P-values were adjusted following Benjamini & Hochberg.

	Variables		DOWN	UP	stat.	df	p_{adj}	signif.
TRIBUTARY	**n**		29	14				
	**Alkalinity**	mg/L	38.0 ±26.2	17.7 ±12.8	3.417	40.96	0.014	*
	**Conductivity**	µS/cm	105.5 ±75.6	51.9 ±33.4	3.225	40.845	0.014	*
	**DIN**	ppb	102 ±61.2	74 ±40.7	1.776	36.609	0.132	ns
	**DOC**	ppb	329.7 ±196.6	421.5 ±224.8	-1.306	22.951	0.251	ns
	**pH**	-	8.3 ±0.4	7.9 ±0.5	2.417	23.435	0.072	ns
	**SRP**	ppb	1.5 ±1.4	1.7 ±1.4	-0.513	26.234	0.649	ns
	**Temp**	°C	8.5 ±4.7	12 ±4.4	-2.353	27.344	0.072	ns
	**Turbidity**	NTU	108.4 ±206.6	37.8 ±32.3	1.796	30.744	0.132	ns
GLACIER-FED	**n**		13	15				
	**Alkalinity**	mg/L	31.0±25.0	17.5 ±19.8	1.569	22.791	0.358	ns
	**Conductivity**	µS/cm	108.9±82.4	73 ±87.2	1.121	25.784	0.501	ns
	**DIN**	ppb	179.3 ±58.9	140.6 ±56.9	1.765	25.161	0.329	ns
	**DOC**	ppb	177.5± 46.9	178.2 ±64.1	-0.037	25.357	0.971	ns
	**pH**	-	8.2 ±0.3	7.7 ±0.6	2.467	22.952	0.237	ns
	**SRP**	ppb	5.7 ±7.3	4.1 v3.2	0.707	15.908	0.674	ns
	**Temp**	°C	4.6 ±2.3	4.5 ±4.0	0.075	22.900	0.971	ns
	**Turbidity**	NTU	991.2 ±691.6	927.5 ±896.2	0.212	25.696	0.971	ns
